# Curing tinnitus with a Cochlear Implant in a patient with unilateral sudden deafness: a case report

**DOI:** 10.1186/1757-1626-2-7462

**Published:** 2009-05-18

**Authors:** Tobias Kleinjung, Thomas Steffens, Juergen Strutz, Berthold Langguth

**Affiliations:** 1 Department of Otorhinolaryngology, University of RegensburgFranz-Josef-Strauss Allee 11, 93053 RegensburgGermany; 2 Department of Psychiatry and Psychotherapy, University of RegensburgUniversitaetsstrasse 84, 93053 RegensburgGermany; 3 Interdisciplinary Tinnitus Treatment and Research Center, University of RegensburgUniversitaetsstrasse 84, 93053 RegensburgGermany

## Abstract

Cochlear implantation is a routine procedure for patients with bilateral profound sensorineural hearing loss. Some reports demonstrated a suppression of tinnitus as a side-effect after implantation. We describe the case of a 55-year-old man suffering from severe right-sided tinnitus in consequence of sudden right-sided deafness. Multiple therapeutic efforts including intravenous steroids and tympanoscopy with grafting of the round window remained unsuccessful. One year after onset of symptoms right-sided cochlear implantation was performed, which resulted in a complete abolishment of tinnitus after activating the implant. Severe unilateral tinnitus after sudden deafness might represent a new indication for cochlear implantation.

## Introduction

Tinnitus is a frequent and often debilitating condition, which is difficult to treat. The most frequently used therapies consist of auditory stimulation and cognitive behavioural treatment aiming at improving habituation and coping strategies. However, more causally oriented therapeutic strategies are lacking and need to be developed to relieve auditory perception disturbances.

Even if the pathophysiology of tinnitus remains incompletely understood there is increasing evidence that tinnitus is related to alterations of neuronal functioning in the central auditory system. Similar like in phantom limb pain, tinnitus as an auditory phantom perception seems to be the correlate of maladaptive attempts of the brain at reorganization due to distorted sensory input. This notion is confirmed by the finding that hearing loss is the most important risk factor for developing tinnitus and that most people with sudden unilateral deafness experience tinnitus. Animal experiments have demonstrated that reduced auditory input causes a dysbalance between inhibitory and facilitatory mechanisms throughout the central auditory pathways, which then results in reorganisation of the tonotopic maps in the auditory cortex. This might represent a neuronal correlate of tinnitus [1]. Accordingly therapeutic strategies that either specifically compensate for hearing loss or normalize auditory input (e.g. hearing aids) have been shown consistently to attenuate tinnitus complaints [2].

Subjects with severe to profound hearing impairment on both ears are considered as good candidates for cochlear implant (CI) surgery. For post-lingual deaf adults a cochlear implant can be a great help in restoring functional comprehension of speech. The prevalence of tinnitus in adult patients, that undergo cochlear implantation for the classical indication of hearing restoration, ranges between 67 and 100% [3]. Improvement of tinnitus complaints after implantation has been reported in many studies [4]. But also worsening of a pre-existing tinnitus or new development of tinnitus after electrode insertion has been described [5]. However documentation and definition of tinnitus complaints in CI patients are often not standardized and therefore difficult to compare to results obtained from classical tinnitus therapies.

Despite extensive research efforts the precise pathogenesis of sudden unilateral deafness remains unclear. Especially in cases with persistent hearing loss patients frequently complain of tinnitus [6]. Here we present the case of a patient with unilateral disabling tinnitus as a consequence of sudden deafness, who was treated successfully with cochlear implantation.

## Case presentation

A 55-year-old Caucasian man from Germany presented in our tinnitus clinic with complaints of severe disabling right-sided tinnitus. The tinnitus resulted from sudden right-sided deafness, which occurred about one year before. In spite of different therapies such as intravenous steroids, infusion of rheological agents and tympanoscopy with grafting of the round window, the affected ear remained deaf. Subsequently, a pre-exisiting mild bilateral tinnitus exacerbated in the right ear (narrow band noise, 5 kHz). Due to the tinnitus the patient complained of insomnia and concentration problems resulting in severe disability of leading a normal life. Pure tone audiometry testing revealed right-sided deafness and moderate hearing impairment on the contralateral side (pure tone average of 500, 1000 and 2000 Hz: 37 dB HL). Electrical promontory stimulation via needle electrode demonstrated normal function of the auditory nerve. Tinnitus masking was impossible due to the right-sided deafness even when sounds were presented to the left ear. Tinnitus severity has been assessed with questionnaires [7,8] and visual analogue scales (VAS) (Table 1). Using a ten-point VAS the tinnitus was rated by the patient according to loudness and annoyance. Analysis of the scores showed severe impairment in consequence of the unilateral tinnitus. Magnetic resonance imaging (MRI) resulted in regular anatomical structures of the cochlea and the cranial nerves. No pathologic processes affecting the brain, the brainstem or the cerebellum were detected.

**Table 1.** Tinnitus assessment before and after cochlear implantation

	TQ-score* (Goebel, 1994)	THI-score** (Newman,1996)	VAS loudness (0-10)	VAS Annoyance (0-10)
Pre-operative	58	66	6	6
1 month post-operative	25	32	1	1
3 months post-operative	4	4	0	0

* Grading according to the tinnitus questionnaire (TQ) [7]: mild = 00-30; moderate = 31-46; severe = 47-59; extreme = 60-84.

** Grading according to the Tinnitus Handicap Inventory (THI) [8]: slight = 0-16; mild = 18-36; moderate 38-56; severe = 58-76; catastrophic 78-100.

The deaf right ear was successfully implanted with a MedEL SONATAti 100 Cochlear Implant (MedEL, Innsbruck, Austria). Post-operative X-Ray revealed a correct position of the electrode in relation to the basal turn of the cochlea. Shortly after activation of the cochlear implant in the context of a standard fitting procedure the patient reported a reduction of his tinnitus, which completely disappeared during electrical stimulation within the following 3 months. With a deactivated implant the tinnitus only reoccurred after presentation to loud noise. The clinical improvement was also reflected by a distinct decrease in the scores of the questionnaires and the VAS (Table 1). Speech reception of monosyllables at 65 dB SPL improved from 0% preoperatively to 60% 3 months after first fitting and the patient reported no conflict of hearing between the implanted and the contralateral ear.

## Discussion

This case report demonstrates complete tinnitus suppression as a consequence of cochlear implantation in a patient with unilateral sudden deafness. The application of cochlear implants for tinnitus relief in patients with unilateral deafness has so far only been described in one study [9]. All 21 patients included in that study had unilateral sensorineural hearing loss accompanied by severe tinnitus for at least two years. In 95% of patients beneficial effects could be demonstrated. Three patients showed complete tinnitus relief like our patient did, whereas the majority demonstrated statistically significant improvement on tinnitus loudness and impact.

According to different pathologic changes that generate neural activity interpreted as tinnitus, there are several possible mechanisms which may account for tinnitus suppression after cochlear implantation. Our report further supports the model of tinnitus pathophysiology, in which chronic tinnitus as an auditory phantom perception might be the correlate of maladaptive attempts at cortical reorganization due to peripheral deafferentation [10]. As a consequence of this theory, restoration of peripheral sensory input may have long-term beneficial effects on tinnitus by plastic reorganization of the central auditory nervous system. Such a mechanism might be reflected by the observed time course in our patient where tinnitus improved over a period of about three months after implantation. Another possible explanation for the positive effect might be the masking of tinnitus following increased auditory information due to the cochlear implant. Residual inhibition might explain tinnitus suppression effects which outlast the active stimulation period for a certain amount of time. But our observation that tinnitus was neither perceived in quiet environments nor during sleep might not entirely account for this theory. The effect of the insertion of the electrode into the cochlear should also be discussed [3]. Cochlear implantation causes immediate and subsequent trauma to remaining cochlear structures. This might be of benefit in patients, in which abnormal activity of hair cells turns out to be a constant trigger mechanism for tinnitus. However, in these patients immediate postoperative effects due to destruction should be expected, which might occur independent from activation of the implant system.

## Conclusion

Summarizing, disabling tinnitus resulting from sudden unilateral deafness should be considered as a new indication for a cochlear implant procedure. As demonstrated in this report and supported by literature data cochlear implantation may represent a chance for complete suppression of tinnitus in selected patients.

## List of abbreviations

CI: Cochlear Implant
MRI: Magnetic resonance imaging
SPL: Sound pressure level
VAS: Visual Analogue Scale
